# “Necessary evil”: the difficulties of establishing programmatic culture in the transfer portal era

**DOI:** 10.3389/fspor.2024.1435321

**Published:** 2024-09-09

**Authors:** Chris Corr, Trevor Bopp, Sarah Stokowski

**Affiliations:** ^1^ Department of Educational & Organizational Leadership Development, Clemson University, Clemson, SC, United States; ^2^ Department of Human Nutrition and Hospitality Management, University of Alabama, Tuscaloosa, AL, United States

**Keywords:** NCAA, college football, organizational culture, coaching, transfer portal

## Abstract

The implementation of the transfer portal and eased transfer restrictions has drastically impacted the migration of college football players. While such athlete autonomy aligns with sweeping organizational policy adopted, and mandated, by the National Collegiate Athletic Association (NCAA), the absence of barriers preventing the mobility of players may have a resultant effect on the development of sustained organizational culture. Through interviews with direct stakeholders currently coaching football at the Football Bowl Subdivision (FBS) level, the findings of this study reveal that while the transfer portal is commonly utilized to achieve short-term success, building a roster composed predominantly of transfer players was perceived as unsustainable in the desired cultivation of meaningful organizational culture. As coaches grapple with the intensified demands to win football games, the findings of this study indicate that sustained long-term programmatic success may be more suitably achieved through the cultivation of distinct organizational culture rather than a reliance on transfer players.

## Introduction

Establishing organizational culture is pivotal for an organization to achieve strategic objectives and maintain its marketplace position, the process for which is facilitated through the communication of organizational values to and between organizational members (1–3). In the context of the National Collegiate Athletic Association (NCAA) Division I Football Bowl Subdivision (FBS), established organizational culture is integral to winning games (4, 5), but increasingly difficult to develop and preserve from season-to-season due to inherent changes among players and coaching staffs. So much so, that historically successful FBS football coaches are often lauded for their ability to establish and maintain winning cultures over extended periods of time (6–8).

Traditionally, coaches have sought to establish and maintain distinct organizational cultures through the recruitment of prospective athletes that fit desired or existent programmatic values (4, 9). When recruiting, coaches initially communicate organizational value systems to prospective athletes (10), but once enrolled, more tenured players communicate entrenched organizational values to their new teammates, further reinforcing acceptable actions and behaviors expected of organizational members. The process becomes cyclical as prospective college athletes that *fit* within the program's culture matriculate through college and perpetuate the sought after norms, beliefs, and attitudes (i.e., organizational culture) by communicating expectations to new members and engaging in behavior to be emulated. In FBS college football, multi-level communication of the value system results in ascription therein, manifesting in a distinct and often palpable organizational culture.

The recent creation of the transfer portal and, subsequently, the NCAA's adoption of eased transfer restrictions have led to drastic changes to the way in which football coaches must consider and plan their recruitment of athletes who will affirm and preserve the current or desired culture. Among NCAA Division I member institutions, 11,902 athletes entered the transfer portal in 2022, representing a 15% increase from the previous year. Comprising the sport with the greatest number of transfers, more than 1,800 FBS football players entered the transfer portal in 2022 (11). During the 2023 season, transfer players made up more than 20% of FBS football rosters, an increase from just 6% in 2019 (12). A recent paradigm shift among FBS football coaches, primarily motivated to win football games (13, 14), prioritizes high-performing players from the transfer portal who can have an immediate impact (15, 16). Although the prioritization of winning games has always been engrained in FBS football, traditionally coaches have relied upon culture construction as a means to build programs to achieve and maintain success over time (4, 6–8). While transfer players may be better adjusted and equipped to instantly contribute to the athletic proficiency of a football program, their reconciliation with current players and incoming recruits, coupled with consistent roster turnover fostered by the normalization of transferring undoubtedly impacts FBS football coaches’ ability to establish and maintain a distinct organizational culture.

As a key component in the maintenance of organizational culture is existent member communication to new members (1), the transfer portal seemingly interrupts this process of communication between players. Given the importance of organizational culture to achieve programmatic goals and on-field success, the normalization of transferring players and the portal itself potentially serve as existential threats to coaches seeking to establish and maintain distinct organizational cultures. As such, the present study sought to examine current FBS football coaches’ experiences with and perceptions of the transfer portal with specific regard to its influence on their ability to establish and maintain a winning organizational culture.

## Review of literature

### Importance of organizational culture

Organizational culture embodies the physical, cognitive, and behavioral values embedded within a unique organizational setting (17). The values embedded within an organization, both tangibly and perceived, serve as guidelines for prescribed behavior among organizational members (18). While distinct, organizational culture is not necessarily static. Rather, organizational culture can fluctuate with evolving organizational demands or objectives (19). Thus, an established culture contributes to an organization's ability to adapt and react to both short- and long-term demands (20). Within unstable institutional settings or dynamic marketplaces, the inherent nature of change and willingness to adapt accordingly are cultural stalwarts of successful organizations (21). In this sense, the environment in which an organization exists dictates, to an extent, the corresponding cultural composition of a given organization (22).

Yet, an organization is inherently a reflection of its members, and accordingly, said members must believe, endorse, and communicate intra-organizationally the mission and values of an organization for culture to formulate, persevere, maintain, and adapt over time (23, 24). In supporting the culture of an organization, existing members perpetuate and communicate expected behaviors, acceptable actions, and ideological beliefs to new members. Considering the necessity of organizational members in establishing and maintaining organizational culture, the importance of recruiting, retaining, and developing individual members is foundational to establishing organizational culture and achieving organizational success (25).

The culture of an organization provides meaning and guidance for members’ actions and behaviors and is an integral component to achieving favorable outcomes (2). In the context of sport, organizational culture is theorized to be a central indicator and determinant of competitive success and maintenance (9). In a traditional sense, establishing organizational culture begins with leadership and authoritative figures, and is most effectively implemented during periods of organizational turnover in which new management groups evaluate existent organizational components to develop contemporary programmatic objectives (26). In competitive sports, these management groups are often members of coaching staffs. For coaches to establish a distinct organizational culture, a core set of values must be communicated to each organizational member (4). This communicated value system must be adequately enforced by coaches for it to be legitimized among organizational members. Disciplinary measures for actions and behaviors not ascribing to the communicated value system serve to further define and reinforce acceptance of the established organizational culture. Although organizational culture is constantly evolving (27), consistent messaging among varying organizational members (i.e., coaches, team captains) serves to maintain the cultivated culture within a given organization. While organizational turnover is commonplace in college athletics (28), culture can be maintained through consistent messaging among existent players and stakeholders.

### Programmatic culture in collegiate athletics

Although organizational culture typically originates with central leadership and managerial figures, culture within a collegiate athletic department is the function of other factors equally salient to leadership. Given the geographic/regional stratification of college athletics in the United States, athletic department often embody cultural competencies similar to the communities in which they exist (29). Schroeder (4, 9) contextualized this impact of community/region on programmatic culture in collegiate athletics as effects based on the external environment. Somewhat correspondingly, Southall and Weiler (30) likened college athletic departments to “company towns” in which the center of industry and everyday life within a distinct community/region was scripted and dictated by an athletic department itself. While such factors pertaining to external impacts based on the community an athletic department is situated are vital to the cultivation of programmatic culture within a collegiate athletic department, ancillary factors pertaining to the institution of college sports in the United States serve to drastically impact programmatic cultures within college athletic departments as well.

In a general sense, the collegiate athletics industry is an industry of competitively driven replication. At the FBS level, athletic departments engage in consistent competition with peer institutions. As each FBS athletic department exists within the same institution setting and abides by similar rules and regulations regarding operation, such constant competition often manifests in the replication of practices deemed successful within the institutional setting itself (28). Such isomorphism is institutionally pervasive within FBS athletics (31, 32) as competing athletic departments compete with one another for similar resources (e.g., fans, multimedia contracts, apparel sponsorships). From a cultural standpoint, such isomorphic formulation is significant within the development of programmatic culture.

Although athletic departments are competing within one another for resources, the structural formation of athletic departments are strikingly similar. From the standpoint of programmatic objectives, athletic departments are resoundingly similar as well (32). Such similarities in structure and directive results in similarities within the organizational cultures of distinct athletic departments as well (33). As FBS athletics as an institutional setting universally values and seeks successful outcomes—both on the field of play and off—successful practices are often replicated among competing peer institutions (28). Accordingly, less successful athletic departments often seek to emulate and replicate successful athletic programs. As athletic programs (i.e., teams) are the aggregated representation of an entire athletic department, it is not surprising such emulation and replication is commonplace among individual teams as well. For instance, less successful football programs tend to hire assistant coaches or coordinators from successful programs in an attempt to replicate such success (28, 34). While replication is commonplace in traditional business settings, the effects on the development of organizational (i.e., athletic department) and programmatic (i.e., team) cultures are nonetheless distinct. Somewhat accordingly given the intensity in which stakeholders prioritize and value success in FBS football, the immediate pervasiveness and programmatic reliance on the transfer portal upon its implementation in 2018 is not surprising.

### Programmatic culture in the transfer portal era

The transfer portal, in combination with eased restrictions on transfer athletes’ eligibility, provides athletes a viable mechanism in which to transfer between NCAA institutional members. Among the various divisions and subdivisions comprising the NCAA, the transfer portal has most drastically affected FBS football programs. In 2022 alone, more than 1,800 FBS football players entered the transfer portal, representing the largest concentration of transfers within any specific NCAA sport (11). Incoming football coaches at the University of Colorado and Texas State University utilized the transfer portal to turnover entire rosters leading into the 2023 college football season (35). While the traditional recruitment of prospective college athletes (i.e., high school athletes) occurs over a months- or years-long period of incubation, transfer portal recruiting takes place annually during one of two designated transfer windows. The abbreviated nature of transfer athlete recruitment makes the determination of organization fit exceedingly difficult for coaches and talent evaluators (35). The transfer portal, somewhat accordingly, has been predominantly adopted in FBS football to improve roster talent, both through targeted attrition and strategic addition.

While FBS football is routinely characterized as a “talent acquisition business” (36, para. 5), the utilization of the transfer portal primarily to improve roster talent affects the embedded culture within an FBS football program. Coaches, constrained by roster size limits and scholarship allotments, can strategically utilize the transfer portal to encourage current players to leave their program (37). As existent players transfer, roster spots open, FBS football coaches target specific players to recruit from the transfer portal in an attempt to improve the talent level of the program. Given transfer athletes have, ostensibly, adjusted to the transitionary difficulties of college and college athletics more adeptly (e.g., strength training, time demands), transfer portal athletes are often viewed as proven commodities relative their high school counterparts (38). Subsequently, the perceived value of transfer players has led programs to organize their recruitment strategies to disproportionately target transfer athletes (39). While talent acquisition is fundamental to college athletics recruiting, the reliance on transfer players at the sake of traditional high school recruitment may hold ramifications for the cultivation of meaningful and successful organizational culture.

Given the perpetuation and maintenance of organizational culture requires stakeholder investment (23) and the consistent communication of values and expectations for behaviors and acceptable actions on behalf of existent organizational members (24), the transfer portal seemingly serves as an existential threat to unique organizational cultures among FBS football programs. While still novel as an area of research given the relative infancy of the transfer portal itself, Aldave (40) contextualized the negative effects of recruiting transfer athletes with regards to the organizational culture of college athletic programs. Acknowledging as much, University of Mississippi (Ole Miss) head football coach, Lane Kiffin, discussed the effect of the transfer portal on organizational culture:

You’re not going to have phenomenal culture. It doesn’t mean I don’t work on it, but I think I have to realize it just is what it is. These transfer kids, they're going to a place if it’s the best at that time. It’s not about the school and they're not in their third, fourth, fifth year with you to where they know how we do it…Unfortunately now it’s like plug and play (41).

As the recruitment, retention, and development of individuals within a given organization is pivotal to establishing a successful organizational culture (25), the prioritization of short-term performance gains through acquiring transfer players comes at the sake of the long-term establishment of a distinct operating culture.

Kiffin's comments regarding culture allude to the prioritization of winning games that pervades the operating procedures of FBS football programs. FBS football coaches, contractually incentivized and socially motivated to win games (42–44), exist within an institutional field wrought with numerous conflicting logics (45). While the stated mission of college athletics is to provide opportunities to prospective college students via athletics and positively contribute to the holistic development of athletes and university communities (46), NCAA athletics take place in a professionalized and commercialized environment that more closely resembles professional sport than amateur athletics (47, 48). While college coaches inherently assume paternal and maternal roles in the lives of athletes (49), one's job security and future career prospects as an FBS football coach are inextricably tied to winning football games. In addition, FBS football coaches exist in a highly volatile field in which results are expected to be accomplished and consistently achieved in a brief amount of time (50, 51). Although establishing and maintaining a distinctive organizational culture is foundational to success in college athletics (9), the dominant and contradictory logics within the institutional setting of NCAA athletics gives credence to the utilization of transfer portal recruiting for short-term performance gains, even at the sake of cultivating a meaningful programmatic culture.

While the transfer portal and eased NCAA restrictions on transfer eligibility have led to the increase and normalization of transferring institutions among the population FBS football players, extant research examining the organizational impact of transfer portal recruitment has been limited (40). As such, the present study examines FBS football coaches’ perceptions of the transfer portal. More specifically, researchers sought to understand the effect of the transfer portal on establishing and maintaining a distinct organizational culture among FBS football programs. Accordingly, the following research questions guided the qualitative approach to this study:

1. To what extent do FBS football coaches prioritize recruiting players from the transfer portal?
2. How does the transfer portal impact FBS football coaches’ ability to establish and maintain distinctive organizational culture?

## Methodology

To examine the perceptions and experiences of FBS football coaches regarding the transfer portal and its corresponding effects on a program's organizational culture, we undertook a methodological approach akin to descriptive phenomenology (52). Given the relative infancy and ongoing normalization of the transfer portal, this method assisted us in objectively gaining insights from the lived experiences of coaches navigating the portal's influence on their programs, and better understand its practical implications from their personal perspectives (53). To this end, semi-structured interviews were utilized to address the research questions in a systematic manner while still allowing for interview fluidity based on participant responses (53).

### Participants

Past research demonstrates the utility of convenience sampling to examine phenomenon specific to NCAA FBS football through the paradigm of individual stakeholders (see 55–57). Thus, the present study implemented convenience sampling to secure interviews with six current FBS football coaches. Subsequent snowball sampling methods (58) resulted in two additional coaches. While eight participants are not enough to produce generalizable findings, the insight gained from the participation of these eight participants was valuable, nonetheless. In fact, a strength of qualitative research is the ability for researchers to more adequately capture the unique and distinct experiences of a more narrow number of participants (59–61). In this sense, the sentiments of the eight participating coaches in this study represent a valuable cross-section of FBS football coaches.

Participants averaged 10 years of coaching experience and represented six different football programs, primarily from Southeast and Midwest states. At the time of the interviews, four of the coaches held positions with institutional members of Autonomy Conferences, while the remaining four worked at Group-of-Five member institutions. The willingness of participants to discuss their perceptions of the transfer portal and programmatic culture is noteworthy considering each coach only agreed to speak with us under the condition of absolute anonymity. To ensure this, and in accord with several of the coaches’ specific requests, we used the broader classification of “assistant coach” rather than disclosing their specific title. Participant demographic information can be found in Table 1.

**Table 1:** Demographics of participants*.*

Pseudonym	Conference affiliation*	Coaching title	Years coaching experience	Former college athlete
Antwan	SEC	Offensive assistant coach	5	No
Charles	Big XII	Defensive assistant coach	8	No
Deshawn	Sun Belt	Defensive backs coach	8	Yes
Doug	ACC	Tight ends coach	34	Yes
John	AAC	Offensive assistant coach	10	No
Martin	Sun Belt	Offensive line coach	5	Yes
Thomas	ACC	Offensive assistant coach	11	No
Winston	Sun Belt	Defensive coordinator	10	No

* Conference affiliation acronyms represent: American Athletic Conference (AAC), Atlantic Coast Conference (ACC), Big XII Conference (Big XII), Southeastern Conference (SEC), Sun Belt Conference (Sun Belt).

### Data collection

A 14-item interview guide (see Appendix A) was developed with insight from three graduate students with multiple years of experience in college football. Questions were designed to address (1) potential differences within the cultures of the schools, programs, and/or organizations at which they worked; (2) personal feelings and experiences regarding the transfer portal; (3) the portal's practical impact on their jobs and programs; (4) the potential necessity of individuals and programs to adjust and revise their recruiting philosophy; (5) lessons learned; and (6) suggestions for improvement and/or adjustment to the NCAA's evolving transfer policies. Interviews were intentionally scheduled in the spring, after the closing of the Winter transfer window, so coaches could reflect on the transfer period they most recently experienced. Interviews took place over Zoom^©^, an online video conferencing platform that enabled us to both record and transcribe interviews. Transcriptions were reviewed and checked against the video recordings to ensure accuracy; after which, data were analyzed.

Semi-structured interviews with participating FBS football coaches were conducted between February and April of 2023 and lasted between 55- and 70-min in length. Interviews with each of the eight participants were recorded and transcribed with all materials pertaining to the data itself stored on a password protected device per institutional review board (IRB) requirements. Transcription was performed manually by two members of the research team. During transcription, all identifiable data that threatened to allude or disclose the identity of participants was removed, anonymizing the data entirely. Such efforts were stated as conditional among each participating coach and ensured by the research team to mitigate participants’ fear of reprisal in their current coaching position.

### Data analysis

As a result of the interview guide's design and subsequent administration, the initial review of transcriptions was inherently deductive in reducing and sorting the data into a more cohesive and manageable dataset (62). Two additional team members then ingratiated themselves with the transcripts and resultant dataset to further code and validate participant quotes into *a priori* themes; thereby creating a framework for readers and researchers alike to engage with the coaches’ voices and lived experiences (63). This process further allowed us to capture the nuance and descriptive nature of the coaches’ exact words (64). As such, the interview data reported in the findings section below consists of the detailed language used by coaches during the interview process.

Transcribed and anonymized interview transcripts were thematically coded by three members of the research team. As such, triangulation was established through the individual coding process and upon comparison of researchers’ notes to establish consensus among coded thematic areas (65). An *in vivo* coding technique was utilized to capture the nuance and descriptive nature of participants’ exact words (64). As such, the interview data reported in the findings section below consists of the detailed language used by participants during the interview process. Inter-coder reliability was established from the outset of the coding process to ensure consistency throughout the coding process (66). Such reliability was established through a group comparison of notes and thematic areas upon the individual initial coding of transcribed data. Each member of the research team conducted this coding and routinely met as a group to discuss pertinent and emergent themes to ensure consistency among coders. In instances where researchers disagreed on the identification of specific codes, the researchers met and established a consensus prior to moving towards a subsequent round of coding. During the initial round of coding, two such instances of disagreement arose and were resolved immediately prior to the second round of coding. During subsequent round of coding, the researchers were consistent and in agreement in their individual identification of codes.

Member checking was utilized by allowing participants to review their transcribed responses. Participants were contacted by the researchers various times until each participant replied the transcribed data accurately represented their opinions and views on the transfer portal. Participants were provided the opportunity to revise statements transcribed in interviews. However, no participants indicated they wished to amend their statements. In addition to member checking, the triangulation of multiple coders analysis to establish consensus among themes contribute to the validity of reported findings.

### Data aggregation and researcher positionality

Given the thematic coding undertaken during this qualitative study and the inherent nature of subjectivity, the disclosure of research positionality is necessary as a component ensuring the reliability of the corresponding data. The three members composing the primary research team—and, correspondingly, the contributing authors—are each presently serving in professorial positions within institutions featuring a Power-5 athletic department. Each of these three researchers has a professional background working in the collegiate athletics industry and remains interconnected within this space. Accordingly, the initial convenience sample of six participants was drawn from the researchers’ connections within the FBS coaching industry.

In an attempt to ensure objectivity and delimit the potential for subjective bias as a result of the researchers’ prior relationships to participants, graduate students at the primary author's current institution were utilized to assist in conducting interviews. Three graduate students were strategically selected based on their experience participating in collegiate athletics, specifically FBS football. Based on these prior experiences, the three graduate students were involved in the preparation and development of the semi-structured interview protocol. As the interview transcripts were anonymized during the transcription process, the primary researchers received interview data devoid of personal identifiers. This was done so strategically as to manufacture a more objective coding procedure. Although the researchers possessed existing relationships with many of the participating coaches and the interviewers had experience competing in FBS football and, subsequently, had experienced the pervasiveness of the transfer portal within the sport itself, appropriate efforts were taken to ensure the validity and reliability of the data presented in this study. It is worth noting that the researchers’ relationships allowed for this study to be conducted and the interviewers experiential understanding of the transfer portal and general setting of FBS football was invaluable in establishing trust and rapport during interviews. While such factors serve as limitations, they were also strengths within this study that we believe added considerable value to the study.

## Findings

Upon completion of the deductive coding process, researchers identified two primary areas in which participants responses were primarily focused: (1) the necessitation of the transfer portal and (2) the effects of the transfer portal. Within both of these primary areas, several emergent thematic areas were present (see Table 2). Accordingly, the following findings section is organized according to these thematic areas with specific regard to the research questions seeking to examine the utilization and impact of the transfer portal on the development of culture.

**Table 2:** Coded thematic areas.

Primary- and sub-themes
Necessitation of the transfer portal
*[sub-theme]* Prioritization of transfer players
*[sub-theme]* Effect of the transfer portal on roster development
Transfer portal's effect on programmatic culture
*[sub-theme]* Cultivating a culture of commitment
*[sub-theme]* Establishing a culture of winning

### Necessitation of the transfer portal

Each coach alluded to the necessity of the transfer portal to accumulate player talent and develop a competitive football roster. Charles referred to recruiting transfer players as “a necessary evil” before going on to discuss the deference afforded to transfer players over prospective college athletes (i.e., high school players) stating, “if there is a very good player in the portal…you are now recruiting him and taking him over a high schooler.” Such preference for transfer players was also indicated by Deshawn, as he stated prioritizing the transfer portal resulted in signing the best players at his respective position in the most recent recruiting class. John confirmed the importance of the transfer portal in accumulating the most talented, and occasionally proven, players in a given recruiting class stating, “a lot of times we aren't getting a great and developed prospect from high school…[the transfer portal] levels the playing field.” Given the return yielded by transfer portal recruiting to date, John stated the prioritization of the transfer portal over traditional high school recruiting had become central to the program's recruiting strategy:

The [transfer] portal has really impacted us and now it has become the philosophy of us taking transfer portal players to be able to build and win. Taking the guys who are wanting a chance and managing the roster.

While not every participant indicated such complete reliance on the transfer portal, the pervasiveness of recruiting transfer players, often at the sake of traditional high school recruiting, was consistent among six of the eight participants.

#### Prioritization of transfer players

The justification for diverting recruiting efforts and shifting strategy to prioritize transfer portal recruiting was universally attributed to the impact of transfer players on team performance. Martin summated the transformative seasonal value of recruiting transfer players:

The [transfer] portal has allowed me to help turn a zero-win team into a team that was close to being bowl eligible the next season and even in reach of beating an SEC [Southeastern Conference] opponent.

Given the importance of winning and sustained competitive success in FBS football, and college athletics in general, the perceived and tangible relationship between recruiting transfer players and winning reinforced many participants willingness to embrace the transfer portal. Antwan crystalized this point:

I am all for taking players in the transfer portal, especially if they will make your team better immediately. Transfers already have the experience that freshmen may be lacking, and you have a better idea of what you are going to get on the field.

Said value in recruiting predominantly transfer players was indicated by multiple other participants at both Autonomy Conference and Group-of-Five affiliated programs. Transfer players, already adjusted to the unique demands and stressors placed on FBS football players, exist as proven commodities. As Charles indicated, a consistent perception among FBS football coaches was that “high school players need more time” than transfer players to significantly contribute and impact the success of the program.

#### Effect of the transfer portal on roster development

The nature of recruiting players from the transfer portal was likened by participants to free agency in National Football League (NFL) professional football. In NFL free agency, organizations bid for the services of players no longer contractually bound to a specific team. Consistent with many recent popular media depictions of FBS football, various participants described the market for transfer players as “The wild, wild West.” Although many participants valued the transfer portal tremendously with regards to talent accumulation and roster construction, several were conflicted considering the significant player attrition they had experienced resulting from the transfer portal. Charles revealed the give-and-take mentality of the transfer portal succinctly:

We are currently experiencing both ends of it. Brought in good players that have really helped this team on and off the field. We also have lost players that would have been patient and worked hard to get their shot.

Antwan, having been retained during the previous season's head coaching transition, discussed the drastic roster turnover that occurred when his program's previous head coach had been fired. However, upon hiring the new head coach, Antwan stated the program “adapted to the times and hit the portal to replace the talent we lost.” While the transfer portal was stated to be a useful tool to establish transformative change in a relative short period of time, the inherent nature of the transfer portal for many FBS programs consists of routine roster attrition season-to-season that is replaced by incoming transfer players. Such consistent organizational turnover has a resultant effect on establishing and developing a distinct organizational culture as existent members depart, and new members consistently arrive.

### Transfer portal’s effect on programmatic culture

#### Cultivating a culture of commitment

Given the consistent roster turnover depicted by participating FBS football coaches and the importance of cultivating and maintaining a distinct organizational culture with regards to sustained programmatic success in FBS football, the coaches’ characterizations of the transfer portal's impact on organizational culture resulted in numerous novel findings. Doug was one of two participants to indicate the transfer portal had not affected his program in any fashion. While the program utilized the transfer portal to accumulate talent and build their roster, Doug alluded to the established philosophy within the program resulting in only utilizing the transfer portal to address specific positional needs:

We aren’t opposed to the [transfer] portal. When the [transfer] portal needs to be used, it will likely be for gaps that may occur in the roster. With consistent good [high school] recruiting classes, the time for [relying on] the [transfer] portal still isn’t up [sic] for our program.

Accordingly, Doug's program prioritizes recruiting prospective college athletes (i.e., high school players) for the expressed purpose of developing players and maintaining the culture embedded within the program. Such culture was reflected in the fact that while his program has had players enter the transfer portal, Doug noted they had not experienced “kids leav[ing] in the middle of the year” and made sure to emphasize that within his program “kids stick out their seasons.” Such statements were made with regards to the established culture unique to the program and the outcomes associated to players’ commitment to the team itself.

While Doug stated his program prioritized developing their roster and embedding a distinct culture among returning players within the organization, Thomas, an offensive assistant coach also in the ACC, stated his program had not recruited a transfer player to date:

We haven’t brought any transfer in yet…just something we talk about and track more preparing for when we might have to utilize it. It will be a really big change to the way we have done things, but we have such a great foundation that we will do it in a way that still represents our program’s core values.

Likewise, Thomas emphasized that his program was not averse to recruiting transfer players but stressed the practice of recruiting a player from the transfer portal did not align with the established culture or values embedded within the football program and, correspondingly, impacted the recruiting strategy utilized by the coaching staff. Thomas's specific acknowledgment of the transfer portal's inevitability is noteworthy considering the pervasiveness of transfer recruiting throughout FBS football. However, the subsequent detail that recruiting transfer players will align with existent programmatic values, a reflection of organizational culture, is indicative of Thomas's program's commitment to a distinct and established culture.

#### Establishing a culture of winning

While these two participants stated that their programs had been indirectly hesitant to embrace the transfer portal due to the perceived threat of roster turnover to unique values embedded within the program, the other six participating FBS football coaches indicated that utilizing the transfer portal was necessary to establish a winning culture. While this winning culture was an organizational commitment that led to heightened expectations of winning football games, multiple participants indicated that continued reliance on the transfer portal after achieving and establishing such objectives was untenable to maintain long-term organizational success. Martin confirmed this point by succinctly stating, “we had to take some transfers to be good this season, but we hope to take a few less every year until we finally get to where we mostly take high school guys.” Such sentiment was also shared by Antwan, who differentiated between the use of the transfer portal for establishing and maintaining success:

The transfer portal is not a key to building a sustainable program. I would prefer to build my program through [high school] recruiting and mix in some transfers here and there. The transfer portal can cause issues with your scholarship numbers if you rely on it too much.

Considering distinct values embedded within programmatic culture are communicated and reinforced by existent members, the obstructive effect of the transfer portal on roster retention (i.e., scholarship numbers) is especially salient.

An additional primary concern of coaches extended beyond winning and roster spots and instead shifted focus to the personal development of FBS football players. Coaches lamented the manifestation of the transfer portal in the mentality that players could readily escape adversity rather than confront it. Winston believed the transfer portal provided “an easy way out for all the players” in which they were “not sticking by your commitment.” While Winston acknowledged there were select instances in which transferring was in the best interest of both the football program and athlete, he described his primary concern as players’ disproportionate pursuit of “where they can go for money or what they think is best in the time.” The emphasis on facing adversity and standing by your commitment to a football program was echoed by numerous other coaches as well. Antwan stated, “the transfer portal can sometimes stray athletes away from the true grind of having to earn your snaps, minutes,” while Martin confirmed, “I hate the idea of how easy it makes it for young people to quit.” Consistent among participants’ responses was the idea that the transfer portal influenced the mentality and personal development of numerous athletes. The outcomes of such effects were discussed by coaches inundated with establishing an organizational culture to counter the resultant psychological effects of the transfer portal on FBS football players.

## Discussion

The participants in this study indicated that the pervasiveness of the transfer portal in FBS football affects the ability of coaches to cultivate longstanding organizational culture. While traditional college athletic programs rely on defined and embedded programmatic value systems that simultaneously embody and become desired organizational culture (4, 9), the committed communication of program value systems is interrupted by the revolving utilization of incoming and outgoing players via the transfer portal. Accordingly, the need for coaches to continuously perform culture (re)construction within their football programs is an additional burden adding to the difficulty in ascertaining and cultivating culture in the transfer portal era. However, while traditional desired programmatic culture has become increasingly challenging, the institutional demands that place the utmost importance on winning in FBS football (42–44) pervades and impacts the decision for football coaches to utilize, and rely on, the transfer portal. Correspondingly, and as indicated by the coaches in this study, while the transfer portal was viewed as an actionable mechanism in which to achieve short-term success (e.g., roster improvement, wins), the continued reliance on the transfer portal was viewed as untenable to sustaining long-term programmatic success and growth.

The immediate impact of transfer players on winning, however, was depicted as a valuable strategy in the pursuit of short-term goals and objectives. The immediacy of the transfer portal to (re)establish and turnover an entire roster was described as a desirable tool for coaches to imprint their unique and evolving desires and ideologies within the program. More specifically, these ideologies were universally described in terms of establishing a winning culture, or a culture that prioritized winning football games above other ancillary factors. In many ways, this prioritization of the transfer portal for the expressed purposes of winning football games aligns with the predominant operating logic permeating the college athletics industry in the United States (47, 48). While such prioritization of winning is not novel in FBS football, the traditional reliance on high school recruits to build a program and embed a desired culture over time has been upended by pressure faced by coaches to achieve immediate results (both recruiting and winning). As indicated by the participants in this study, the reliance on the transfer portal to form or maintain a competitive roster can have resultant effects on the ability for coaches to formulate a desirable programmatic culture.

Given the realities of NCAA policy and regulations that FBS football programs operate within, the prioritization of recruiting transfer players marginalizes the recruitment of high school players as well. Traditional culture development within college athletics relies on the successful recruitment of high school athletes that learn, assimilate, and perpetuate a distinct organizational culture during their collegiate athletics career (4, 9, 10). The prioritization of the transfer portal, regarding roster development, alters this traditional process in which organizational culture is fostered and disseminated. While college football players possess a variety of unique motives when selecting an institution to enroll (67, 68), the coaches’ experiences demonstrate the pervasiveness of the transfer portal resulting in a greater emphasis on the transactional nature of the recruiting process. Given the influx of name, image, and likeness (NIL) compensation and the value of college athletic performance to professional athletic opportunities, the ability for college athletes to transfer freely has resulted in more salient and economically driven recruiting circumstances (69). While football players are undoubtedly pursuing transfer opportunities for their personal benefit (70, 71), coaches are also utilizing transfer players to quickly achieve individual objectives as well. The indication that such a strategy prioritizing transfer players is unsuitable for long-term success lends credence to the value of sustained organizational culture development for the purposes of achieving strategic objectives beyond the short-term. While transfer players may immediately impact a team, coaches in this study viewed the transfer portal as a deterrent toward high school recruits’ opportunities to develop within the program while establishing a culture that emphasizes winning as of vital importance. In this sense, and as a practical application, the reliance on the transfer portal is perhaps valuable to coaches during transitionary periods but detrimental to overall coaching longevity.

The utilization of transfer players to achieve short-term goals (i.e., winning games), and in the process forsaking long-term programmatic culture development, is a byproduct of the demands placed on FBS football coaches. Typically, and historically, roster turnover has been a systematic process requiring multiple contiguous recruiting classes. Accordingly, FBS football coaching staffs require multiple years to overhaul a roster through high school recruiting and achieve successful outcomes (i.e., win football games) (13, 72, 73). In the transfer portal era, however, relatively instantaneous success is more readily achievable and expected through the pursuit of transfer players. More adept physically and mentally to the demands of college football, transfer players provide coaches an opportunity to remake rosters over the course of months rather than years. Given the importance of football success in the business of college athletics (42), the expectation for coaches to achieve immediate results corresponds with programmatic utilization of the transfer portal in lieu of traditional high school recruiting. In addition, as transfer players make increasingly transactional decisions when determine their enrollment intentions (69), the difficulty of maintaining an organizationally rooted roster is more challenging than any previous era of college football.

For Group-of-Five programs, such a challenge is perhaps heightened by the pervasiveness of the transfer portal, making year-to-year retention of players increasingly difficult. As Group-of-Five transfer players prioritize opportunities for professional development and financial gain (74), the allure of competing at the highest level of college football entices them to pursue transfer options. As such, the parity between FBS subdivisions grows increasingly less with the onset of the transfer portal. As Group-of-Five programs experience success, contributing players are perhaps likely to pursue transfer to Autonomy Conference programs for the purpose of exposure, NIL, and level of competition. Accordingly, Group-of-Five programs are potentially predisposed to consistently rely on the transfer portal as players’ ambitions influence their decision to enroll at Autonomy Conference programs. Such transfer outcomes assuredly affect the ability for coaches at each subdivisional level to cultivate meaningful organizational culture given the revolving nature of roster turnover now nearly omnipresent in FBS football, a sentiment shared by the coaches we worked with.

Conversely, the use of the transfer portal was indicated by coaches to positively affect outcomes pertaining to winning football games. Accordingly, Group-of-Five coaches are also perhaps simultaneously hampered by the loss of players to Autonomy Conference programs and bolstered by the addition of transfer players formerly enrolled at Autonomy Conference programs. This migration of players within the subdivisional level of the FBS is of potential expressed benefit to coaches as they seek career advancement. As the coaches indicated, the utilization of the transfer portal to achieve immediate success has been embraced during coaching staff transitions, specifically at the Group-of-Five level as well. As a matter of praxis, such strategy could be of strategic advantage for Group-of-Five coaches seeking career advancement to the Autonomy Conference level. Given that Autonomy Conference athletic administrators seek out younger coaches and afford them more time to succeed than older coaches (75), the ability for Group-of-Five coaches to utilize transfer players to achieve immediate success may manifest in greater advancement and lucrative coaching opportunity at the Autonomy Conference level. While winning games affords coaches more benefits of an upward career trajectory, the lack of programmatic culture cultivation through the traditional recruitment and development of high school football players may be of a disservice to coaches’ career longevity upon transition to Autonomy Conference programs.

## Conclusion

The findings of this study detail the effects of roster turnover on the cultivation and maintenance of culture among FBS football organizations. Coaches stated transfer players, as with high school recruits, must adjust and conform to embedded program values and norms for culture to sustain over time. Such a process is of greater difficulty due to the consistent threat of player attrition. As the loss of players to the transfer portal results in the loss of entrenched members to communicate the culture itself, the effect of the transfer portal on organizational culture development is two-fold: (1) the loss of veteran leadership, and (2) lack of roster continuity year-to-year. Although prioritizing the transfer portal was stated to be a viable means of establishing a distinct culture predicated on the importance of winning, continued reliance on the transfer portal long-term was explicitly stated by participants to be detrimental to the maintenance of such culture. This dichotomy between the importance of winning and the cultivation of programmatic culture is noteworthy considering the operating logics of NCAA athletics, and more specifically FBS football.

Given the importance of winning and organizational success in the institutional setting of the NCAA (42, 47, 48), the prioritization of football players via the transfer portal aligns with the intense pressure to win athletic competitions. As institutional theory, and the corresponding logic(s) within an institutional setting, provides a mechanism to understand the process of change in organizational culture (76), the pervading logics fundamental to the operation of the NCAA serve to influence FBS football coaches in their pursuit of athletes in the transfer portal at the sake of organizational culture. Given the perceived transformative value of transfer players and the emphasis placed on winning in FBS football, the prioritization of transfer portal recruiting aligns with these unique operating logics of NCAA athletics. However, deviant outcomes can arise among organizations solely prioritizing winning athletic competitions (77). Given the increased transactional nature of college football recruiting, and college athletics writ large, the potential for organizational deviancy resulting from the hyper-pursuit of winning games to meet the structural demands and ideological operation of NCAA athletics may foster deleterious outcomes for players and coaches alike, as well as college football programs and the greater institution.

## Limitations & future research

Given the nature of the convenience sample relied upon in gathering participants for this study, an inherent limitation exists in the manner data was accumulated. While the authors disclosed their positionality pertaining to the initial convenience sample and utilized various methods to ensure the objective presentation of findings, the sample utilized in this study remains a limitation worth reporting. In addition, the findings of this study are not generalizable or able to be inferred on a larger scale of FBS coaches. While the nature of qualitative research is not to achieve generalizable findings but rather uncover and interpret the experiences of participants within a larger setting, it is notable to mention that the effects of the transfer portal are undoubtedly unique depending on a multitude of factors.

Although a limitation, these factors present an opportunity for future scholarly inquiry. Specifically, future research examining the effects of the transfer portal on FBS programs in either Power-5 or Group-of-5 conferences would be valuable. In addition, future research examining the outcomes associated with prioritizing transfer portal recruiting from both football performance and organizational culture standpoints would be valuable both practically and theoretically as college coaches and administrators continue to adjust to the changing legislative structure of the NCAA. In addition, research to determine the effect of the prioritization of the transfer portal on coaching mobility is necessary to further understand the impact of the transfer portal on coaches’ career trajectory and programmatic culture at the Group-of-Five level.

## Data Availability

The original contributions presented in the study are included in the article/Supplementary Material, further inquiries can be directed to the corresponding authors.

## Appendix A

Semi-Structured Interview Guide

Background Questions

1. What school are you currently coaching or affiliated with?
2. How long have you been coaching or affiliated with the school you are currently at?
3. How many total years have you worked in athletics (any level)?
4. Are you a former athlete? (If yes) What are some key differences within the coaching culture as you have made the transition from player to coach. (If no) What made you want to work with athletics?

Content Questions

5. What are the main differences within the cultures of the schools, programs, and/or organizations you have been a part of? What are some differences that have occurred over the years of your tenure?
6. Can you describe your feelings regarding the Transfer Portal?
7. Has the transfer portal affected your job and you individually?
8. Has the transfer portal impacted your university and program?
9. What are your personal thoughts, and your team's philosophy, on recruiting transfers over high school prospects?
10. Has the transfer portal impacted recruiting and scholarship numbers your program takes?
11. Is the transfer portal ‘here to stay” and/or what are the next steps that you see on the horizon?
12. Is there anything you have learned through the process of recruiting transfers that you are going to start to do or implement?
13. In what ways can the transfer portal be improved?
14. Is there anything else you'd like to say regarding the transfer portal?
